# Static Electricity as a Curative Agent—Report of Cases

**Published:** 1898-06

**Authors:** N. A. Olive

**Affiliations:** Waco, Texas


For the Texas Medical Journal

### Static Electricity as a Curative Agent—Report of Cases.

BY N. A. OLIVE, M. D., WACO, TEXAS.

[Read before the Waco Medical Association, February 8, 1898.]

In dealing with static electricity as a curative agent it may not be expected to cure all cases in which it is apparently indicated. It is not a specific. But as to its value as a remedial agent, as to its usefulness in doing that which all other remedies have failed to do, as to its analgesic effect, as to its worth as a restorative in an overworked condition of body and mind, as to its power to induce sleep, as to its serviceableness in exciting normal action of the secretory and excretory organs, as to its value in atonic dyspepsia and chronic catarrhal gastritis, as to its ability to impart sensation and motion to the paralytic, and as to its inestimable value as a general tonic, to speak conservatively, it takes rank with any remedy known to the medical profession.

In the employment of this agent any medicinal agent which may be indicated need not be discontinued. It can be used in conjunction with almost any other form of treatment.

About 104 A. D., Galen and others spoke of the therapeutic effect of the shock of the electrical fish, a case of gout having been cured by this means. It was also recommended in various forms of neuralgia. Since that time electricity has been employed in one form or another, until now it occupies the front among remedies of unquestionable merit. The physiological effect of electricity in its various forms and its indication in the treatment of disease has been fully demonstrated, and it is now as well understood as any remedy with which we have to deal.

But as to functional electricity. In dealing with disease the conscientious practitioner gives a remedy of known physiological effeet, the dose (barring idiosyncracies) having been demonstrated by use.

You do not permit the substitution of an inferior article for the genuine. You do not subject a critical case to untried remedies when you have those at your command that you can rely upon. Neither do you give a remedy of reputed value a half trial, fail to get the desired result and condemn it as worthless. So it is with static electricity,—it would not be just to apply it to a patient who had gone the rounds, taken almost every known remedy for a variously diagnosed disease without benefit, and because it failed to afford relief in this case condemn it as utterly worthless and cast it aside forever. But it will not be of infrequent occurrence that static electricity, persisted in, will fail to disappoint you even in this class of cases. One point right here: You will find among the laity the idea of electricity possessed by many is a lightning flash, the heavens ablaze, a clap of thunder and all is over. Or a street car flying by, a crackling and sparkling and on she goes. They think whatever electricity will do for them, it will do quick. 'Tis true the relief will sometimes be so prompt as to astonish even the experienced physician; but it is not always the case. The patient must first be prepared to take a continued course of treatment. His mind must be relieved of the expectation of lighting-like results. Again, occasionally a patient will not tolerate it well, and short, mild treatments will at first be necessary until tolerance is established. But above all insist that after your patient gets to feeling pretty well he should not discontinue treatment too soon.

Of first importance is a good generator. An inferior machine may as justly be compared to a good one as may an inferior drug to the genuine. With a good machine it will be the exception that you will find a day that it fails to generate all right, whereas with an inferior one it will be the exception for it to work to do any good. The large Morton, Wimhurst or Holtz machine is regarded as first-class by those having them. The most serious objections to these machines is the price, which is out of the reach of many general practitioners, and the fact that it is run with a crank, necessitating the presence of a third person when a treatment is given. In point of construction it seems almost perfect, so far as I am able to judge. And the objectionable crank can be done away with by the additional cost of a motor. There are other good machines, but an extended description of any machine would not be in taste tonight, as this body appreciates practical results more than descriptive literature. One point may very well be borne in mind: do not imagine that every machine encased in glass to afford protection against atmospheric changes is a good one, for there are some terrible frauds encased in glass. The machine made by Dr. Gawne, of Sandusky, Ohio, with which you all are familiar, is, in my opinion, as good for all practical purposes as any made. It runs with a treadle, doing away with the necessity of a third person to run the generator. With care as to cleanliness of the machine and to keep the room closed during damp weather, it will generally do its work well. My experience of more than two years with it has been very satisfactory, there having been few days in which it failed to generate well.

Having desisted from any description of the various machines so also will I relieve you of the necessity of listening to a dry historical account of electricity as a remedial agent, and will present the report of a few cases in which you may feel some interest.

Case 1.—Mr. H., age 24, had a severe attack of typhoid fever. Was sick eight or ten weeks. Recovery slow, but all right except motor paralysis from knees down, unable to walk. Appetite poor and digestion feeble. Began treatment about three months after his recovery from the fever. Not much emaciation except below the knees, where there seemed to be nothing but skin and bones. Applied static spark, positive pole over affected parts and spine and stomach. Fifteen minutes' treatment. From first treatment he began to eat more, digestion improved and felt that he was gaining strength. On the fourteenth day took measurement of calves of legs and found the muscular tissues had developed to the extent of three and a half inches increase in circumference. He could now walk by holding onto the wall, by which means he could go up and down stairs. At the end of six weeks he returned home, walking fairly well, and continued to improve. Treatment was given daily. It is now two years since he was dismissed, and he is still doing well.

Case 2.—J. P., a teacher; age 26; insomnia. Has always been a hard student. Present condition extremely nervous. Says he gets but two or three hours of extremely nervous sleep during the night. Generally lies awake until 3 or 4 o'clock in the morning and gets up at 6. Applied static breeze to head and cervical region.

1st night.—No improvement.

2nd night.—Was not so restless, but slept about the usual time.

3rd night.—Got about seven hours of quiet sleep; no nervousness. He said he slept like a child.

Continued treatment ten days, when he was entirely relieved of both insomnia and nervousness. This was eighteen months ago, and he has had no return of the trouble.

Case 3.—Mr. G., farmer and stockraiser; sciatica. Has suffered six months, gradually growing worse. Greatly emaciated. Does not sleep more than three or four hours a night, and that is induced by morphia. When he began electrical treatment all other treatment was stopped. Applied static spark, positive pole, over sciatic nerve fifteen minutes, breeze over head five minutes. Daily treatment. Not any apparent improvement until the fifth night, when he slept well seven or eight hours. From this time nervous symptoms began to disappear. Never had another sleepless night. Pain gradually diminished until the twenty-fifth day, when he returned home free from pain and increased in weight and strength. This was eighteen months ago, and he has had no trouble since.

Case 4.—Mrs. H., widow, age 25; milliner. Painful menstruation and hysteria. Menstruation irregular. As time approaches within a day or two of the flow the pain in back and ovarian region becomes almost unbearable, and convulsions come on. Began treatment immediately after cessation of flow, Applied positive spark over ovarian region and spine ten minutes, breeze over head five minutes. Daily treatment until next period, which came on without her knowledge. No pain whatever, no convulsions, flowed more freely and lasted four days; whereas previously it had been scant and lasted generally about two days, never more than three. Dismissed her with instructions to return in about three weeks for daily treatment. Treated her a week before each period for three months. It is more than a year since she was finally dismissed, and there has been no return of the trouble.

Case 5.—Miss S., age 25. Painful menstruation with scanty flow. Periods regular, of three days' duration. Positive spark over ovarian and sacral regions for two weeks prior to period. Daily treatment. Result—flow free enough, four days' duration, no pain. Returned for treatment three days before next two periods. No trouble whatever. Will go three or four months without pain, and then feels some uneasiness, and comes for a treatment, then has no further trouble for three or four months again.

Case 6.—Miss S., age 22. Amenorrhœa, with great pain and extreme nervousness. Positive spark over ovarian and sacral regions ten minutes, breeze over head and spine five minutes. Daily treatment for one month. No relief experienced. Would not submit to examination. Soon afterwards married and was examined. Had stenosis of cervix. This was corrected with relief of symptoms. Had she submitted to examination at first the stenosis could have been relieved with the uterine electrode and galvanic current and she would not have had any further trouble.

These few cases will illustrate the value of static electricity in general medicine. I could have presented some cases of ague, some of atonic dyspepsia with bilious diarrhœa, some of severe forms of facial and intercostal neuralgia, cases of lumbago, etc., with as happy results as any of the foregoing. Some cases of gout with enlarged joints, deformed hands and feet, stiffness, etc., treated and cured by the application of static electricity and correction of diet. In gouty cases, of course, many are incurable, but I have failed to find one whose symptoms have not been greatly modified by this treatment. This paper is already rather long, but one point may well be borne in mind: any other treatment indicated can be employed in connection with electricity. The foregoing cases had no other treatment.

